# Pulse oximetry signal loss during hypoxic episodes in preterm infants receiving automated oxygen control

**DOI:** 10.1007/s00431-024-05549-9

**Published:** 2024-04-09

**Authors:** Lukas O. Langanky, Karen B. Kreutzer, Christian F. Poets, Axel R. Franz, Christoph E. Schwarz

**Affiliations:** 1 Department of Neonatology, University Children’s Hospital, Tübingen University Hospital, Tübingen, Germany; 2 Center for Pediatric Clinical Studies, University Children’s Hospital, Eberhard Karls University Tübingen, Tübingen, Germany; 3 Department of Neonatology, Center for Pediatric and Adolescent Medicine, University of Heidelberg, Im Neuenheimer Feld 430, 69120 Heidelberg, Germany

**Keywords:** Automated oxygen control, Signal loss, Pulse oximetry, Preterm infant

## Abstract

The aim of this study was to analyze signal loss (SL) resulting from low signal quality of pulse oximetry-derived hemoglobin oxygen saturation (SpO_2_) measurements during prolonged hypoxemic episodes (pHE) in very preterm infants receiving automatic oxygen control (AOC). We did a post hoc analysis of a randomized crossover study of AOC, programmed to set FiO_2_ to “back-up FiO_2_” during SL. In 24 preterm infants (median (interquartile range)) gestational age 25.3 (24.6 to 25.6) weeks, recording time 12.7 h (12.2 to 13.6 h) per infant, we identified 76 pHEs (median duration 119 s (86 to 180 s)). In 50 (66%) pHEs, SL occurred for a median duration of 51 s (33 to 85 s) and at a median frequency of 2 (1 to 2) SL-periods per pHE. SpO_2_ before and after SL was similar (82% (76 to 88%) vs 82% (76 to 87%), *p* = 0.3)).

*  Conclusion*: SL is common during pHE and must hence be considered in AOC-algorithm designs. Administering a “backup FiO_2_” (which reflects FiO_2_-requirements during normoxemia) during SL may prolong pHE with SL.

*  Trial registration*: The study was registered at www.clinicaltrials.gov under the registration no. NCT03785899.

**Table:** 

**What is Known:** *• Previous studies examined SpO2 signal loss (SL) during routine manual oxygen control being rare, but pronounced in lower SpO2 states.* *• Oxygen titration during SL is unlikely to be beneficial as SpO2 may recover to a normoxic range.*	
**What is New:** *• Periods of low signal quality of SpO2 are common during pHEs and while supported with automated oxygen control (SPOC), FiO2 is set to a back-up value reflecting FiO2 requirements during normoxemia in response to SL, although SpO2 remained below target until signal recovery.* *• FiO2 overshoots following pHEs were rare during AOC and occurred with a delayed onset; therefore, increased FiO2 during SL does not necessarily lead to overshoots.*

**What is Known:**

*• Previous studies examined SpO2 signal loss (SL) during routine manual oxygen control being rare, but pronounced in lower SpO2 states.*

*• Oxygen titration during SL is unlikely to be beneficial as SpO2 may recover to a normoxic range.*

**What is New:**

*• Periods of low signal quality of SpO2 are common during pHEs and while supported with automated oxygen control (SPOC), FiO2 is set to a back-up value reflecting FiO2 requirements during normoxemia in response to SL, although SpO2 remained below target until signal recovery.*

*• FiO2 overshoots following pHEs were rare during AOC and occurred with a delayed onset; therefore, increased FiO2 during SL does not necessarily lead to overshoots.*

## Introduction

Pulse oximetry–derived hemoglobin oxygen saturation (SpO_2_) is standard of care in neonatal intensive care units for monitoring gas exchange and controlling oxygen titration. We conducted a study comparing two different algorithms of an automated oxygen controller (AOC, “SPOC” algorithm, Fritz Stephan Gmbh, Gackenbach, Germany) with routine manual control (RMC), also evaluating two different averaging times (2 s vs 8 s) [1]. In line with previous studies [3–7], we found an increased proportion of time spent in the predefined SpO_2_-target range during AOC and a reduction of hypoxemic episodes (HE) compared to RMC alone [1, 2]. As prolonged hypoxemic episodes (pHEs) were recently identified as the major physiologic predictor of severe bronchopulmonary dysplasia and death and are known to be associated with impaired long-term neurodevelopment, preterm infants might benefit from reduced exposure to pHEs [8, 9]. Interestingly in our study, for a median (range) of 5% (3 to 7%) of the time during 2-s SpO_2_ averaging and 1% (1 to 4%) during 8-s averaging, the signal quality was reduced in the controlling pulse oximetry signal (signal loss, SL) while infants received AOC with the current SPOC algorithm [1]. SL has been previously evaluated in a study conducted by Lim et al. where it was more common at low SpO_2_ values [10]. They suggested that blind FiO_2_ adjustments are not beneficial during SL. In line with this recommendation, all currently available AOC algorithms, to our knowledge, postpone automatic oxygen titrations during SL. Specific for the SPOC algorithm, the FiO_2_ is set to a pre-defined back-up value typically reflecting FiO_2_-requirements during normoxemia. When good signal quality resumes, automated FiO_2_ control is re-enabled according to the current SpO_2_. As these responses to SL may affect the performance of AOC during pHE, we aimed to investigate the occurrence of SL during pHE with the current SPOC algorithm.

The main objective of this analysis was to investigate the frequency and duration of SL as well as SpO_2_ before and after such periods. Secondary outcomes included timing and duration of desaturation and subsequent hyperoxemic “overshoots.”

## Methods

This was a post hoc analysis of an unblinded randomized crossover study of SPOC [1]. In short, 24 preterm infants were analyzed with a GA < 34 weeks receiving respiratory support including supplemental oxygen. Patients were randomized to five different sequences (routine manual control (RMC), the new SPOC algorithm and the old SPOC algorithm, while SpO_2_ averaging time was set to 2 s or 8 s) with 6-h recordings at each averaging time—SPOC combination. The new algorithm differed from the old by enabling to set an upper limit for FiO_2_ (in our study set to 0.15 above baseline FiO_2_), faster decrements to above baseline FiO_2_ after normalization of SpO_2_ following an hypoxemic event and slower decrements to below baseline (see [1] for a detailed description). For this analysis, we evaluated the AOC periods with the current algorithm (SPOC new).

A bedside pulse oximeter (Radical7, Masimo SET, Irvine, USA) was used for controlling the SpO_2_ signal which fed its signal into the ventilator where the SPOC algorithm was implemented (Sophie, Fritz Stephan Gmbh, Gackenbach, Germany). The SPOC algorithm then adjusted the FiO_2_ according to the current SpO_2_. During SL, specifically the SPOC algorithm was programmed to set the current FiO_2_ to a preset backup value. In our study, back-up-FiO_2_ was set to follow “baseline FiO_2_” where the latter is a value that can be set manually, but is also updated every 5 min while AOC is enabled to asymptotically approximate the mean actual FiO_2_ of the preceding 15-min time interval. (which typically reflects FiO_2_ requirements during normoxemia). Therefore, in case of an increased FiO_2_-demand during a pHE, SL is resulting in a reduction of FiO_2_ due to being set back to the preset backup-value. As soon as the SpO_2_-signal resumed, SPOC would titrate FiO_2_ according to the current SpO_2_.

Episodes of pHE (episodes with SpO_2_ saturations < 80% for ≥ 30 s) and their duration (SpO_2_ dropping below 90% until returning to the target range (SpO_2_ at 90 to 95%) for at least 8 s) were analyzed for the presence of SL and their characteristics. We also evaluated differences in number and duration of SL between both averaging times (2 s vs 8 s). Hyperoxemic “overshoots” were defined as SpO_2_ ≥ 97% for a minimum of 8 s within 120 s after recovery of SpO_2_ to the target SpO_2_ range.

### Statistical analysis

Categorical variables were described using frequencies and percentages, continuous variables using medians and interquartile ranges (IQRs). For comparisons between SL groups (yes, no), the Mann–Whitney *U* test was used for continuous variables, and the *χ*^2^ test or Fisher’s exact test (in case of small expected counts) was used for categorical variables. All analyses were performed using IBM SPSS Statistics (version 28, IBM Corp, Armonk, NY, USA). All tests were two-sided and a *p* value < .05 was considered statistically significant.

## Results

**Fig. 1:**
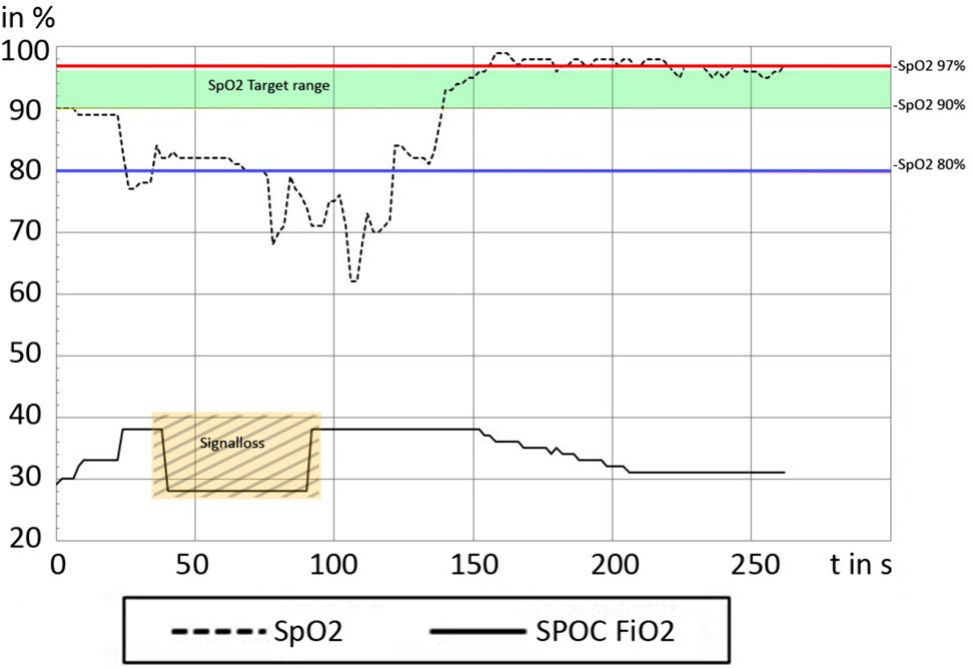
Prolonged hypoxic episode example. Hyperoxia threshold: SpO_2_ 97%; hypoxemia threshold SpO_2_ = 80%;  SpO_2_ target range (90 to 95%); dotted line: patients SpO_2_; black line: set FiO_2_ from SPOC; barred box = signal loss defined as low signal quality

Demographic details have been published previously [1]. In short, 24 participants receiving non-invasive respiratory support including supplemental oxygen (median (IQR) gestational age at birth 25.3 weeks (24.6 to 25.6)) were evaluated. The duration of the evaluated recordings with new SPOC was 12.7 h (12.2 to 13.6 h) per infant in which we identified 76 pHEs with duration of 119 s (86 to 180 s) in 22 (92%) participants. In three (13%) participants, no SL occurred during pHEs, whereas in 19 (79%) participants, at least one SL period was identified within pHEs (13 (68%) were found to have pHEs with and without SL). In total, 102 episodes of SL occurred in 50 (66%) pHEs (See Fig. 1 for an example, Table 1 for details of pHEs). SpO_2_ before and after SL did not differ significantly (82% (76 to 88%) vs 82% (76 to 87%), *p* = 0.3). SpO_2_ after SL were either below (*n* = 90 (88%)), within (*n* = 11 (11%)) or above target (*n* = 1 (1%)).

**Table 1:** Characteristics of prolonged hypoxemic episodes with and without signal loss

**Variables**	**SL group**		
	**SL**	**No SL**	***p* value**
Frequency			
Number of pHE	50 (66%)	26 (34%)	
Number of pHE per patient	3 (1 to 4)	2 (1 to 3)	
Number of pHE under 2 s averaging	30 (60%)	10 (39%)	
Number of pHE under 8 s averaging	20 (40%)	16 (61%)	

Duration (s)			
Total pHE duration (s)	135 (112 to 228)	81 (58 to 116)	< .476^a^
Time with SpO_2_ < 80% (s)	62 (44 to 91)	43 (34 to 72)	< .005^a^

Signal Loss			
Number of SL periods	102 (100%)	N.A	
Number of SL periods per pHE	2 (1 to 2)		
Absolute Time per pHE (s)	51 (33 to 85)		
Relative Time per pHE (%time)	40 (18 to 61)		
SpO_2_ at SL Start (%)	82 (76 to 88)		
SpO_2_ at SL End (%)	82 (76 to 87)		

Overshoots within 120 s following recovery			
Number of periods	8 (16%)	8 (31%)	.244^a^
Absolute duration (s)	58 (24 to 82)	35 (23 to 45)	.244^a^
Relative duration (% of 120 s)	48 (20 to 68)	29 (19 to 38)	.740^a^
Delay after recovery (s)	14 (9 to 21)	16 (4 to 61)	

Data are presented as median (quartile 1 to quartile 3) or *n* (%). Overshoots are defined as SpO_2_ ≥ 97% for at least 8 s within 120 s after reaching the SpO_2_ target range following a pHE; SPO_2_ in %

*SL* signal loss defined as low signal quality or missing values, *pHE* prolonged hypoxemic episode defined as SpO_2_ < 90% until recovery to SpO_2_ ≥ 90% for at least 8 s including a hypoxic period (< 80%) of at least 30 s duration, *N.A.* not applicable

^a^From Mann–Whitney *U* test

When comparing the averaging times of 2 s vs 8 s, pHE with SL was more common during 2 s averaging (60% vs 40%), but the difference was not statistically significant (*p* = 0.770). The median duration of SL periods was 20 s (14 to 36 s) vs 32 s (12 to 52 s; *p* = 0.547).

Hyperoxemic overshoots occurred in 22 pHEs, of which six were due to manual override.

## Discussion

In this post hoc analysis of prospectively recorded data, we showed that SL is common during pHEs. SL occurred in 2/3 of pHE-episodes and lasted for approximately 40% of the pHE-duration, compared to the previously reported proportion of total study time of 5% (3 to 7%) with SL during 2 s averaging and 1% (1 to 4%) with SL during 8 s averaging in the total study time [1]. Lim et al. reported that the proportion of SL time was increased in lower SpO_2_ ranges (< 85%) compared to higher SpO_2_ ranges (> 95%) [10]. We focused on even lower SpO_2_ values (< 80%). Comparing the different averaging times, we saw no significant difference in SL duration or its frequency of occurrence.

As periods of SL lead to a halt in automatic oxygen titration, our findings are relevant to the design of AOC algorithms in general. Specifically for the SPOC algorithm, FiO_2_ is reduced by falling back to the preset “back-up FiO_2_” which likely affects controller performance during the pHE. Under manual FiO_2_ control, Lim et al. suggested that blind FiO_2_ adjustments are unlikely to be of benefit, and the SpO_2_ reading for SL-episodes starting in hypoxia (SpO_2_ < 85%) returned at a median value that was 3.2% higher than at the start of SL [10]. According to our data in SL-episodes starting in hypoxemia, SpO_2_-readings returned to about the same SpO_2_ level thus were still in a rather hypoxic range. Of note, the recovery in SpO_2_ resulted in values below target in 90/102 SL-periods (88%), whereas SpO_2_ above target was identified in a single episode only (1/102 SL-episodes (1%)). Therefore, the vast majority of SL-episodes started and ended below target. We interpret this finding as indicating only a low risk of overshoot hyperoxemia in case FiO_2_ would have been higher during SL. We interpret this finding as indicative of a low risk of overshoot hyperoxia in case FiO_2_ would have been higher during SL. The time spent with SpO_2_ < 80% was significantly higher in pHEs with SL vs pHE without SL; furthermore, pHE duration was longer in case of SL. Even though our sample was smaller, it points to the fact that “blind” decrements in FiO_2_ to the backup-value due to the implemented SPOC algorithm potentially prolong HEs. We suggest that these automatic reductions in FiO_2_, by returning to a back-up FiO_2_ mostly influenced by normoxic periods during pHEs while SL occurs, are of questionable benefit. An adoption of the algorithm would mean to disable these automatic reductions in FiO_2_ during pHEs and would maintain increased FiO_2_ while the infant remains hypoxemic. Furthermore, even slight FiO_2_ increments might be considered during SL. This would potentially further shorten HE duration but might increase the risk of overshoot-hyperoxemia after recovery (although unlikely, considering that SpO_2_-readings returned to the hypoxic range).

Therefore, a prospective clinical evaluation of every adaptation of AOC-algorithms is mandatory.

### Limitations

Being a post hoc analysis, validity of our study might be reduced. However, our analysis is based on objective parameters and prospectively recorded data. Generalizability is limited, as this was a single-center, small study that only included patients on non-invasive respiratory support.

## Conclusion

SL during pHE is common and should be considered when designing an AOC algorithm. AOC, in our case SPOC, responding to SL by falling back to “back-up FiO_2_” might prolong the duration of pHE because SpO_2_ before and after SL did not differ in our study. With the SPOC-algorithm, overshoots after SL were uncommon and with delayed onset and, therefore, maintaining an increased FiO_2_ during SL in pHE might not increase overshoots.

## Data Availability

Data available on resonable request.

## Abbreviations

AOC: Automated oxygen control
FiO_2_: Fraction of inspired oxygen
GA: Gestational age
HE pHE: Hypoxemic episode prolonged hypoxemic episode
SL: Signal loss (periods with low signal quality)
SpO_2_: Oxygen saturation
SPOC: AOC algorithm (Fritz Stephan GmbH)
